# Construction of a Multisite DataLink Using Electronic Health Records for the Identification, Surveillance, Prevention, and Management of Diabetes Mellitus: The SUPREME-DM Project

**DOI:** 10.5888/pcd9.110311

**Published:** 2012-06-07

**Authors:** Gregory A. Nichols, Jay Desai, Jennifer Elston Lafata, Jean M. Lawrence, Patrick J. O’Connor, Ram D. Pathak, Marsha A. Raebel, Robert J. Reid, Joseph V. Selby, Barbara G. Silverman, John F. Steiner, W.F. Stewart, Suma Vupputuri, Beth Waitzfelder, on behalf of the SUPREME-DM Study Group

**Affiliations:** Jay Desai, Patrick J. O’Connor, HealthPartners Research Foundation, Minneapolis, Minnesota; Jennifer Elston Lafata, Henry Ford Health System, Detroit, Michigan; Jean M. Lawrence, Kaiser Permanente Southern California, Pasadena, California; Ram D. Pathak, Marshfield Clinic, Marshfield, Wisconsin; Marsha A. Raebel, John F. Steiner, Kaiser Permanente Colorado, Denver, Colorado; Robert J. Reid, Group Health Research Institute, Seattle, Washington; Joseph V. Selby, Patient Centered Outcomes Research Institute, Washington, DC; Barbara G. Silverman, Maccabi Healthcare Services, Tel Aviv, Israel; W.F. Stewart, Geisinger Health System, Danville, Pennsylvania; Suma Vupputuri, Kaiser Permanente Georgia, Atlanta, Georgia; Beth Waitzfelder, Kaiser Permanente Hawaii, Honolulu, Hawaii. Dr Elston Lafata is also affiliated with Virginia Commonwealth University, Richmond, Virginia.

## Abstract

**Introduction:**

Electronic health record (EHR) data enhance opportunities for conducting surveillance of diabetes. The objective of this study was to identify the number of people with diabetes from a diabetes DataLink developed as part of the SUPREME-DM (SUrveillance, PREvention, and ManagEment of Diabetes Mellitus) project, a consortium of 11 integrated health systems that use comprehensive EHR data for research.

**Methods:**

We identified all members of 11 health care systems who had any enrollment from January 2005 through December 2009. For these members, we searched inpatient and outpatient diagnosis codes, laboratory test results, and pharmaceutical dispensings from January 2000 through December 2009 to create indicator variables that could potentially identify a person with diabetes. Using this information, we estimated the number of people with diabetes and among them, the number of incident cases, defined as indication of diabetes after at least 2 years of continuous health system enrollment.

**Results:**

The 11 health systems contributed 15,765,529 unique members, of whom 1,085,947 (6.9%) met 1 or more study criteria for diabetes. The nonstandardized proportion meeting study criteria for diabetes ranged from 4.2% to 12.4% across sites. Most members with diabetes (88%) met multiple criteria. Of the members with diabetes, 428,349 (39.4%) were incident cases.

**Conclusion:**

The SUPREME-DM DataLink is a unique resource that provides an opportunity to conduct comparative effectiveness research, epidemiologic surveillance including longitudinal analyses, and population-based care management studies of people with diabetes. It also provides a useful data source for pragmatic clinical trials of prevention or treatment interventions.

## Introduction

For many years, diabetes registries have been used to assess and enhance clinical care provided by health systems (1). Initially, diabetes registries were created from administrative data such as inpatient diagnoses and pharmaceutical dispensing data (2,3). Other early registries included insurance claims data that may have been incomplete because patients could receive care from multiple uncaptured sources (4). The more recent availability of detailed clinical data, including real-time laboratory test results, has improved the ability to build more sophisticated and accurate diabetes registries that capture more precisely defined cohorts of people with diabetes.

The Agency for Healthcare Research and Quality (AHRQ) recently published a user’s guide for creating and using patient registries (5). However, implementation of standardized methods for creating multisite diabetes registries and validating their accuracy is largely untried. Standardization of methods is especially important when data are aggregated and patterns of care are compared in diverse populations across multiple health systems (6). Many single-site studies have validated administrative definitions of diabetes (4,7-11), but criteria for inclusion in registries often vary, rendering comparisons across systems difficult. Such differences are not trivial; Harris and colleagues showed that in a sample of residents of Ontario, Canada, diabetes prevalence ranged from 5% to 12%, depending on which combinations of laboratory, pharmacy, and diagnosis data were used to verify diabetes case status (12).

A standardized method for identifying people with diabetes from the detailed clinical information available in electronic health records (EHRs), applied across multiple health systems, would be a powerful tool for conducting comparative effectiveness research, monitoring trends, analyzing geographic variation, and conducting surveillance of prediabetes and diabetes. The objectives of this study were to identify people with diabetes from comprehensive EHR and administrative data of 11 integrated health systems and to estimate incident cases of diabetes among them.

## Methods

### Participating health systems

The SUrveillance, PREvention, and ManagEment of Diabetes Mellitus (SUPREME-DM) project is an AHRQ-funded study under the PROSPECT (Prospective Outcome Systems using Patient-specific Electronic data to Compare Tests) initiative that brings together a consortium of 33 diabetes researchers (Appendix) from 11 of the 18 member organizations of the HMO Research Network (HMORN). Health plans participating in SUPREME-DM include 6 Kaiser Permanente regions (Northern California, Southern California, Northwest [Oregon/Washington], Hawaii, Colorado, and Georgia), as well as HealthPartners (Minnesota), Marshfield Clinic (Wisconsin), Geisinger Health System (Pennsylvania), Group Health Cooperative (Washington), and Henry Ford Health System (Michigan). These 11 health plans had approximately 10 million enrollees in 2009.

### Virtual data warehouse

During the past 10 years, the HMORN has developed a Virtual Data Warehouse (VDW) with initial support from the National Cancer Institute. The VDW is now an HMORN resource supported by member organizations and network consortia and has been described in detail elsewhere (13). Briefly, the VDW is a data model resulting from the efforts of 8 working groups, each focused on a specific type of data (eg, laboratory results, pharmacy, enrollment), that map site-specific data to a common standard. Each site maintains an operating and accessible version of the standardized data. Within each research center of the member systems, data extracted from confidential health plan databases are reconfigured into 14 core data tables using standard variable names and values. The data can be linked through a unique patient identifier common across tables. The VDW, from which the diabetes DataLink is constructed, enables use of standardized data extraction programs distributed to all participating sites. Each site constructs individual-level data sets for analysis and sends either aggregated or individual-level deidentified data sets to the lead site, where they are combined into overall comparable cohorts.

The VDW data are more comprehensive than the claims data available to commercial insurers, Medicare, or Medicaid because the VDW tables include extensive data from EHRs as well as administrative data. Nine of 11 SUPREME-DM participating systems use an EPIC-based EHR system (EPIC, Verona, Wisconsin). VDW tables include comprehensive laboratory results that are useful for establishing risk-factor levels (eg, glycosylated hemoglobin, fasting and random plasma glucose, serum lipids) and blood pressure, height, and weight measurements, all of which can be used to assess the presence and level of hyperglycemia, dyslipidemia, hypertension, and overweight/obesity. In all participating health plans, at least 90% of members have a pharmacy benefit that helps ensure near complete identification of drug dispensings.

### Protection of human subjects

Each of the 11 participating health system–affiliated research organizations has its own institutional review board (IRB). Under a common review mechanism (14), the IRBs of 8 sites ceded review authority for construction of the DataLink to a ninth participating system (Kaiser Permanente Colorado), and 2 retained local IRB oversight. Because the DataLink currently consists of aggregate secondary observational data and holds only minimal risk of unauthorized disclosure of protected health information, we requested and received waivers of informed consent and Health Insurance Portability and Accountability Act (HIPAA) authorization. Nonetheless, we used several safeguards to minimize the possibility of breaches of confidentiality. Data sent to the lead site were aggregated so that reconstructing observations on any single member in the analyses was impossible. These aggregated data were transferred to the lead site via a secure data transfer website that meets HIPAA and Medicare data security standards; every keystroke and all attached files were encrypted.

Future studies may require limited data sets for ancillary analyses, primary data collection, or implementation of interventions on samples of study participants drawn from the DataLink. Such studies will require separate IRB review and approval. Collaborating sites can always choose whether to participate in future studies and will decide whether to cede or retain review by their local IRB.

### Identification of SUPREME-DM DataLink population

For the SUPREME-DM project, we identified all 15,765,529 members of the respective health systems, regardless of age, who had any enrollment (membership) from January 1, 2005 (the first year that all systems had complete EHR data), through December 31, 2009. The use of health plan members rather than all patients who received care in the delivery system allowed us to establish an unbiased denominator for calculation of population rates. Because we sought to capture all prevalent cases of diabetes and the earliest source of identification for incident cases, we searched for all available data from January 1, 2000 (the first year VDW data were available), through December 31, 2009, to create a series of dichotomous indicator variables that could be used to identify possible diabetes, while retaining the dates and values of the qualifying indicators. These included inpatient or outpatient diagnoses of diabetes, laboratory test results conducted in an outpatient setting that were diagnostic of diabetes, and pharmaceutical dispensings.

### Identification of members with diabetes

Using the indicator variables, we applied an algorithm to estimate the number of members with diabetes (Table 1). For criteria comprising 2 elevated laboratory values from the same test, we required the tests be performed on separate days but no more than 2 years apart. Two outpatient diagnoses were also required to occur on separate days. We excluded members with only a metformin, exenatide, or thiazolidinedione dispensing who met no other criteria to ensure that women with polycystic ovary syndrome or members with prediabetes were not included. To avoid including women with gestational diabetes, indicators were not considered during periods of pregnancy, defined as delivery date minus 270 days. The resulting data set is a dynamic cohort that can be linked to all available VDW data, thus allowing for longitudinal analyses of people with diabetes. Because many people join their health system after diabetes has been diagnosed, the first recognition of diabetes cannot be assumed to be the first date of diagnosis, and the source of diabetes identification for these people has little meaning. For this report, we calculated years of enrollment with diabetes from the first date associated with an indication of diabetes until December 31, 2009, or the last date of health system eligibility, whichever came first.

**Table 1:** SUPREME-DM DataLink Project Criteria for Identifying Members With Diabetes

Criterion	Value	Details
Dispense of sulfonylurea, insulin, biguanide, thiazolidinedione, α-glucosidase inhibitor, incretin mimetic, meglitinide, amylin analog, or dipeptidyl peptidase inhibitor	≥1 Dispense	Not valid if dispense was for metformin, any thiazolidinedione, or exenatide **AND** none of the other criteria were met
**OR**		
HbA1c	≥2 at ≥6.5%	Tests must be on separate days no more than 2 years apart.
**OR**		
Fasting plasma glucose	≥2 at ≥126 mg/dL	Tests must be on separate days no more than 2 years apart.
**OR**		
Random plasma glucose	≥2 at ≥200 mg/dL	Tests must be on separate days no more than 2 years apart.
**OR**		
Random plasma plus fasting glucose	1 at ≥200 mg/dL **AND** 1 at ≥126 mg/dL	Tests must be on separate days no more than 2 years apart.
**OR**		
HbA1c plus fasting glucose	1 at ≥6.5% **AND** 1 at ≥126 mg/dL	Tests can occur on same day but cannot be more than 2 years apart.
**OR**		
HbA1c plus random plasma glucose	1 at ≥6.5% **AND** 1 at ≥200 mg/dL	Tests can occur on same day but cannot be more than 2 years apart.
**OR**		
2-h 75-g OGTT	1 at ≥200 mg/dL	Do NOT count if measured during pregnancy.
**OR**		
Inpatient discharge diagnosis	≥1 (250.x, 357.2, 366.41, 362.01–362.07)	Primary or secondary.
**OR**		
Outpatient visit diagnosis	≥2 (250.x, 357.2, 366.41, 362.01–362.07)	Visits must occur on separate days. Ambulatory visits only. Do not include telephone, e-mail, emergency department, laboratory, radiology, or other encounter types.

Abbreviations: HbA1c, hemoglobin A1c; OGTT, oral glucose tolerance test.

### Identification of incident diabetes

We considered a member to have incident diabetes if the first indication of diabetes followed at least 2 years of continuous enrollment in a health system with no other indication of diabetes. For these members, we considered the date associated with the first indication of diabetes to be equivalent to a diagnosis date, and years of enrollment with diabetes to be equivalent to diabetes duration, calculated as time between diagnosis date and end of health system membership or December 31, 2009, whichever came first. Because our earliest EHR information was from 2000, by definition, our earliest incident cases were identified in 2002. Thus, although we can identify incident cases, we cannot estimate a true diabetes incidence *rate* from these data because requiring 2 years of eligibility before diabetes identification creates a subset of members with different inclusion criteria than the total population, and thus different denominators.

## Results

### Size of the population with diabetes

The 11 systems contributed 15,765,529 unique members over the 5-year period, of whom 1,085,947 (6.9%) met the SUPREME-DM DataLink criteria for diabetes (Table 2). On average, these members had 5 years of health system membership following diabetes identification. Mean age at diabetes identification (55.7 y) and the proportion who were women (48.1%) were consistent across systems.

**Table 2:** Total Health Plan Enrollment and Number of Members With Diabetes, by Centers Participating in the SUPREME-DM Project, 2005-2009

Site	Enrolled Members, n	Members With Diabetes, n (%)	Mean Years of Enrollment After Diabetes Diagnosis^a^	Mean Age at First Diabetes Indication, y	% Female
1	836,169	51,989 (6.2)	5.1	56.6	48.7
2	4,904,140	361,894 (7.4)	5.4	56.5	48.0
3	305,968	20,331 (6.6)	5.3	59.4	52.0
4	5,488,817	389,647 (7.1)	5.0	55.8	47.0
5	568,157	31,842 (5.6)	4.3	52.9	49.2
6	224,821	27,781 (12.4)	5.6	58.1	52.5
7	220,934	26,680 (12.1)	4.6	60.7	49.7
8	1,463,011	61,212 (4.2)	4.8	54.6	47.1
9	392,858	30,066 (7.7)	5.4	56.8	52.2
10	577,495	42,926 (7.4)	5.1	57.1	51.0
11	783,159	41,579 (5.3)	5.0	57.7	47.6
Total	15,765,529	1,085,947 (6.9)	5.0	55.7	48.1

^a^ Calculated as number of years following first indication of diabetes as described in Table 1 until end of health system enrollment or December 31, 2009, whichever came first.

Of the members identified as having diabetes, 39.4% (n = 428,349) were incident cases, for whom we were able to estimate a date of diagnosis and who had a mean of 3.3 years of membership following diagnosis (Table 3). Overall, 9.9% of incident cases were first identified from inpatient diagnoses, 22.6% from outpatient diagnoses, 20.7% from pharmaceutical dispensings, and 46.8% from outpatient laboratory test results. There was considerable variability between systems in how incident cases were recognized. For example, the proportion first identified by laboratory tests ranged from 17.8% to 67.3%.

**Table 3:** Number of Incident Diabetes Cases, Years of Membership Following Diagnosis Date, and Source of First Indication of Diabetes, by Health System, 2002–2009

Site	Incident Diabetes Cases^a^	Mean Years of Enrollment With Diabetes^b^	Source of First Indication of Diabetes^c^			
			Inpatient Diagnosis, %	Outpatient Diagnoses, %	Pharmacy Dispense, %	Outpatient Laboratory Results, %
1	18,287	3.3	11.0	17.5	16.6	54.9
2	151,177	3.6	9.6	20.0	20.1	50.2
3	7,248	3.3	14.4	36.2	12.4	37.0
4	153,473	3.4	9.8	18.9	23.2	48.1
5	10,149	3.2	15.0	26.7	26.8	31.5
6	12,062	3.5	6.1	41.4	17.0	35.5
7	8,253	2.7	16.6	40.5	11.9	31.0
8	27,096	3.1	4.3	51.2	26.7	17.8
9	11,832	3.5	8.5	13.7	10.5	67.3
10	15,059	3.3	14.2	19.0	16.3	50.5
11	13,713	3.4	12.7	16.9	16.0	54.5
Total	428,349	3.3	9.9	22.6	20.7	46.8

^a^ Number of diabetes cases with at least 2 years of health system eligibility before first indication of diabetes.

^b^ Calculated as number of years following first indication of diabetes until end of health system enrollment or December 31, 2009, whichever came first.

^c^ Based on study criteria in Table 1, the source of the earliest indication of diabetes.

## Discussion

The SUPREME-DM project has united a large consortium of researchers with extensive expertise in childhood, adult, and gestational diabetes to identify more than 1 million unique individuals with diabetes from comprehensive EHR and administrative data of 11 integrated health systems, of whom 428,349 had incident diabetes. Because the DataLink is constructed from comprehensive inpatient, outpatient, pharmaceutical dispensing, and laboratory results data available from the EHR, clinical, and administrative databases of each of these health care systems, and because these data are extracted from defined populations with a known denominator, the DataLink is a unique resource for conducting comparative effectiveness research, surveillance, and epidemiologic studies of unprecedented scale and clinical detail.

Use of registries has enhanced medical care for patients with diabetes in individual health care systems for 2 decades (1). Indeed, several of the participating sites were early developers of diabetes registries derived from electronic data (2,3,11,15,16) and have used these registries for clinical care, quality improvement, and research purposes. However, these registries have traditionally been limited to patients served by only 1 health care delivery system, and variation in how registries were created has impeded cross-system comparisons (9). One major goal of the SUPREME-DM DataLink is to standardize data definitions across participating systems to provide the best possible estimates of diabetes and its complications. The variability among organizations in the proportion of people with diabetes and the source of recognition of incident cases emphasizes the need for this next step.

Although useful for more limited analyses, other previous or existing electronic registries cannot provide equivalent data for analysis. For example, in conjunction with the Centers for Disease Control and Prevention, a collaboration of 3 managed care organizations developed a unified system in 1998 for conducting diabetes surveillance, tracking health services, and delivering preventive care (17). That system has not been maintained. The Department of Veterans Affairs (VA) has an excellent linked national database of VA patients that has been used to identify patients with diabetes, but the population is not representative because patients are predominantly male and sicker than the overall population of patients with diabetes (8). One diabetes database recently developed by the University of Pittsburgh Medical Center (UPMC) represents a more heterogeneous population, combining data from a large number of insurers (18) but covering only a single region. It is unclear whether the UPMC database will be routinely refreshed or whether a denominator of patients with and without diabetes can be easily identified, an essential component for estimating rates of diabetes and its complications. After assessment of other US diabetes databases, we believe the SUPREME-DM DataLink is unique in its size, comprehensiveness, and geographic coverage.

Currently, the best estimates of US adult diabetes prevalence emerge from analyses of the National Health and Nutrition Examination Survey (NHANES). Those data suggest that 7.7% of the US adult population (aged ≥20 y) had diabetes in 2005–2006 (19). Similarly, we found that 6.9% of all enrollees (including children) in the SUPREME-DM DataLink have diabetes. NHANES identifies people with diabetes on the basis of self-report and by a single, unconfirmed elevated laboratory test result. Our DataLink has much more robust parameters to confirm diabetes status. Furthermore, as a cross-sectional survey, NHANES can estimate diabetes prevalence but not diabetes incidence. The longitudinal nature of the DataLink will allow the estimation of the incidence of diabetes and its complications, a unique feature that holds promise for future research and national surveillance efforts.

As recently noted by the Institute of Medicine (IOM), no surveillance system operates nationally and in a coordinated manner to integrate current and emerging data (20). The IOM report calls for a system that includes data on incidence and prevalence over time, primary and secondary prevention (including early detection), health outcomes following surveillance, representative samples, and disparities, noting that EHR data will play a key role in the surveillance of chronic disease. The SUPREME-DM DataLink answers that call by using the actual medical records of more than 15 million people. The comprehensive EHR data available to the DataLink can be used to conduct population-based studies of the complications of diabetes while accounting for a wide range of demographic and clinical characteristics that independently contribute to risk. Furthermore, by examining data before and after diabetes diagnosis, the SUPREME-DM DataLink can be used to study the complete natural history of hyperglycemia and its associated complications.

Despite our standardized definition of diabetes, we observed variation across sites in how members with incident diabetes were initially identified. In addition to differences in the demographic makeup of the site-specific populations, there are several possible explanations. Although each of the 11 sites participating in SUPREME-DM is an integrated health care delivery system, their organizational structures differ (even across the 6 Kaiser Permanente regions). Furthermore, laboratory tests may not use the same reference ranges in all sites, and the use of hemoglobin A1c (HbA1c) assays, although moving toward standardization, could introduce variation. Differences in how providers code diagnoses during outpatient encounters or the inclusion of diagnostic codes linked to laboratory procedures or prescriptions could also introduce variation in the identification of diabetes across sites. Incomplete data capture at some sites, specifically of laboratory tests conducted outside the system or prescriptions filled outside of system pharmacies, could also contribute to variation. Site differences in ascertainment may lead to apparent but artificial differences in diabetes duration or severity — a topic for future SUPREME-DM research. These possibilities are all under investigation. Despite these potential sources of variation in diabetes identification across sites, however, it is likely that a patient with diabetes in any of the systems will be recognized in a reasonably short period of time, especially when multiple data sources such as pharmacy, diagnosis codes, and laboratory results are used for this purpose. Indeed, approximately 85% of diabetes cases in all sites had multiple indications.

As with any observational data collected for health care and payment, there are potential limitations to the SUPREME-DM DataLink. Inconsistencies in data availability (eg, not all sites can distinguish between random and fasting glucose tests) may preclude use of the DataLink for certain purposes or require exclusion of some participating centers from specific analyses. Unrecognized or unmeasurable differences among our study sites in the use of EHRs and the completeness of data could lead to inaccuracies and potential bias in the estimation of diabetes incidence and prevalence. The patient populations in integrated health delivery systems may not generalize to patients managed in less integrated settings, in other geographic areas, or to uninsured populations. A common case identification algorithm was used to identify members with diabetes across all SUPREME-DM sites, but we did not have the resources to individually validate each case through medical record review. Thus, ancillary studies should use caution when approaching individual health plan members because of the occasional member with a coded diagnosis who may not truly have diabetes. An additional limitation is the inability to distinguish members with type 1 and type 2 diabetes with a high level of precision. Finally, date of diabetes diagnosis, an important element in analyses of the natural history and clinical outcomes of diabetes, is not known for 60% of the diabetes cases.

We are expanding the DataLink to include members at risk for developing diabetes on the basis of elevated fasting glucose, glucose tolerance, or HbA1c tests that do not meet diagnostic criteria for diabetes, and to identify women with gestational diabetes. Data for additional years (2010–2012) will be added as they become available. The SUPREME-DM DataLink is a valuable resource that provides an opportunity to conduct comparative effectiveness research, epidemiologic surveillance including longitudinal analyses, and population-based care management studies of people with diabetes, gestational diabetes, and prediabetes, and to explore associated risk factors, complications, and health outcomes in new ways. The DataLink also provides an excellent source for pragmatic clinical trials of preventive or treatment interventions to improve the health and quality of care for people with diabetes.

## Appendix: Research Teams by Site

Kaiser Permanente Colorado (lead site): Christina Clarke; William (Troy) Donahoo, MD; Glenn Goodrich; Andrea R. Paolino, MA; Marsha Raebel, PharmD; Emily Schroeder, MD, PhD; Michael Shainline; John F. Steiner, MD, MPH; Stan Xu, PhD

Group Health Cooperative: Lora Bounds; Gabrielle Gundersen; Katherine Newton, PhD; Robert Reid, MD; Eileen Rillamas-Sun, PhD

Geisinger Health System: Brandon Geise; Ronald Harris, MD; Rebecca Stametz, MPH; Walter (Buzz) Stewart, PhD, MPH; Xiaowei (Sherry) Yan, PhD

Henry Ford Health System: Nonna Akkerman; Liz Dobie, MPH; Jennifer Elston Lafata, PhD; Aida Li; Heather Morris; Abraham Thomas, MD, MPH

Health Partners: Mary Becker; Jay Desai, MPH; Patrick O’Connor, MD, MPH; Kris Ohnsorg, RN, MPH; Nancy Sherwood, PhD

Kaiser Permanente Hawaii: Ameena Ahmed, MD, MPH; Cynthia Nakasato, MD; John Parker; Beth Waitzfelder, PhD; Rebecca Williams, DrPH, MPH

Kaiser Permanente Northern California: Cathy Chou, MPA; Assiamira Ferrara, MD, PhD; Andy Karter, PhD; Romain Neugebauer, PhD; Joe Selby, MD; Julie Schmittdiel, PhD; Bix Swain

Kaiser Permanente Northwest: Brian Hazlehurst, PhD; Teresa Hillier, MD, MS; Terry Kimes; Eric Kopp; Stephen Kurtz; Gregory A. Nichols, PhD; Daniel Sapp

Kaiser Permanente Southern California: Jean Lawrence, ScD, MPH, MSSA; Melissa Preciado; Jian (Leon) Zhang; Chengyi Zheng, PhD

Kaiser Permanente Southeast: Melissa Butler, PharmD, MPH, PhD, BCPS; Ashli Owen-Smith, PhD; Junling Ren; Douglas Roblin, PhD; Suma Vupputuri, PhD

Marshfield Clinic: Amit Acharya, BDS, MS, PhD; Aaron Miller; Ram Pathak, MD; Luke Rasmussen; Trish Siegler, MBA

Maccabi Health System: Anthony Heymann, MD; Barbara Silverman, MD, MPH

Johns Hopkins University: Jodi Segal, MD, MPH

University of Michigan: Michele Heisler, MD, MPA
